# Optical and Piezoelectric Study of KNN Solid Solutions Co-Doped with La-Mn and Eu-Fe

**DOI:** 10.3390/ma9100805

**Published:** 2016-09-28

**Authors:** Jesús-Alejandro Peña-Jiménez, Federico González, Rigoberto López-Juárez, José-Manuel Hernández-Alcántara, Enrique Camarillo, Héctor Murrieta-Sánchez, Lorena Pardo, María-Elena Villafuerte-Castrejón

**Affiliations:** 1 Instituto de Investigaciones en Materiales, Universidad Nacional Autónoma de México, Ciudad Universitaria, A.P. 70-360, México D.F. 04510, Mexico; jesusalejandropea@gmail.com; 2 Departamento de Ingeniería de Procesos e Hidráulica, Universidad Autónoma Metropolitana-Iztapalapa, A.P. 55-534, México D.F. 09340, Mexico; fgg@xanum.uam.mx; 3 Unidad Morelia del Instituto de Investigaciones en Materiales, Universidad Nacional Autónoma de México, Antigua Carretera a Pátzcuaro No. 8701, Col. Ex Hacienda de San José de la Huerta, Morelia C.P. 58190, Mexico; rlopez@iim.unam.mx; 4 Instituto de Física, Universidad Nacional Autónoma de México, A.P. 20-364, México D.F. 04510, Mexico; josemh@fisica.unam.mx (J.-M.H.-A.); cgarcia@fisica.unam.mx (E.C.); murrieta@fisica.unam.mx (H.M.-S.); 5 Instituto de Ciencia de Materiales de Madrid (ICMM), CSIC. c/ Sor Juana Inés de la Cruz, 3. Cantoblanco, Madrid 28049, Spain; lpardo@icmm.csic.es

**Keywords:** perovskite, alkaline niobates, co-doping, lead-free ceramics, Rietveld method, EPR, optical activity, dielectric permittivity, ferroelectricity, piezoelectricity

## Abstract

The solid-state method was used to synthesize single phase potassium-sodium niobate (KNN) co-doped with the La^3+^–Mn^4+^ and Eu^3+^–Fe^3+^ ion pairs. Structural determination of all studied solid solutions was accomplished by XRD and Rietveld refinement method. Electron paramagnetic resonance (EPR) studies were performed to determine the oxidation state of paramagnetic centers. Optical spectroscopy measurements, excitation, emission and decay lifetime were carried out for each solid solution. The present study reveals that doping KNN with La^3+^–Mn^4+^ and Eu^3+^–Fe^3+^ at concentrations of 0.5 mol % and 1 mol %, respectively, improves the ferroelectric and piezoelectric behavior and induce the generation of optical properties in the material for potential applications.

## 1. Introduction

Lead-based piezoelectric ceramics, such as the lead titanate-zirconate (PZT) family, have played an important role for several decades because of their excellent piezoelectric and electromechanical properties [1]. However, due to the use of PbO during processing and its toxicity, the development of lead-free piezoceramics has become a worldwide research topic in materials [2,3,4]. Alkali niobate ceramics based on K_0.5_Na_0.5_NbO_3_ (KNN) have received more attention after Saito et al. [5] improved the piezoelectric coefficient d_33_ to 416 pC/N by doping with Li, Ta and Sb, making the perovskite more covalent and increasing the <001> orientation.

The (Na_1−x_K_x_)NbO_3_ (KNN) is a solid solution formed between an antiferroelectric compound NaNbO_3_ and a ferroelectric compound KNbO_3_, whose phase diagram shows that, at room temperature, the orthorhombic phase becomes stable in the range of 0.475 < x < 1, and the monoclinic phase becomes stable in the range of 0.32 < x < 0.475 [6,7,8]. In the KNN processing the main disadvantage is the volatility of sodium and potassium at temperatures above 850 °C used at synthesis and sintering steps [9].

It has been reported that addition of Mn_2_O_3_ or MnO_2_ could be used to reduce the leakage current in KNN ceramics, because they diminish the amount of oxygen vacancies formed due the volatilization of alkali ions by increasing the valence of Mn^2+^ or decreasing the valence of Mn^4+^ [10,11,12]. Besides, this selection of doping is promising as transparent glass ceramics showed optical properties ascribed to Mn^2+^ ions [13].

On the other hand, lead-free ((1 − x)/2) K_0.5_Na_0.5_Nb_0.95_Ta_0.05_O_3–x_/2Fe_2_O_3_ piezoelectric ceramics (x = 0, to 3.0 mol %) have been prepared by conventional ceramic sintering process [14]. K^+^ and Na^+^ vacancies, originating from the volatilization of alkali metal elements, and charge compensation by the diffused Fe^3+^ ions at low doping concentration (0.4 mol %) might be responsible for the higher tetragonal distortion of the structure and for inducing domain wall motions, which improves the electrical properties. Fe_2_O_3_ has been added also to enhance the piezoelectric properties in single crystals of ferroelectric potassium tantalate niobate (KTa_x_Nb_1−x_O_3_) [15].

Previous reports have shown that, additionally to the improvement of the piezoelectric properties, luminescent response can be achieved by doping KN and KNN with rare-earths, such as Pr^3+^, Er^3+^ and Eu^3+^ [16,17,18,19]. Rare-earth doped materials have been of great scientific interest in developing photonic devices and for the next flat-panel display generation. Eu^3+^-doped materials have been extensively studied because of their transitions from ^5^D_0_ to ^7^F_J_ (J = 0, 1, 2, 3, 4) levels. Mainly, the transition ^5^D_0_ to ^7^F_2_, involved in wavelength peaks around 610–630 nm, shows bright red luminescence and laser action in a variety of glasses and single crystals [16,17,18].

Being a ferroelectric with perovskite structure KNN ceramics have been widely studied as the host material for photoluminescence application in nonlinear optics. The doping is of great importance in modifying the crystallographic phase and tuning photoluminescence properties in the invisible-near infrared range [16,17,18]. Although not yet studied, it is possible that co-doped KN and KNN could exhibit simultaneously piezoelectric and luminescent properties. The works on simultaneous characterization of the electrical (ferro- or piezo-electric) and optical properties in the search for multifunctional materials are scarce to date but slowly increased in the last few years [20,21,22]. Besides, co-doping for this dual purpose has not yet been studied.

This work reports the synthesis of KNN and its solid solutions substituting Na^+^ and K^+^ in the A site of perovskite structure with La^3+^ and Eu^3+^, and Nb^5+^ in the B site with Mn^4+^ and Fe^3+^. The co-doped studied materials were prepared according with the formulations of ((K_0.5_Na_0.5_)_(1−(4x/5)_)La_(4x/5)_)(Nb_(1−(4.5x/5))_Mn_(4.5x/5)_)O_3_ and ((K_0.5_Na_0.5_)_1−x_Eu_x_)(Nb_1−x_Fe_x_)O_3_, with x = 0.005 and x = 0.01. The purpose of using ions in those pairs was to maintain the charge balance and to establish the co-dopant effect avoiding redox process induced by vacancies generation. Furthermore, transition metals were added with the aim to enhance the ferroelectric, piezoelectric and dielectric properties [12,14,23], meanwhile both Mn and rare earths cations are expected to arise optical activity and La is expected to minimize the manganese reduction [10,11,12].

## 2. Results and Discussion

### 2.1. Structural Characterization

In order to facilitate data handling, the chemical formulas of the studied compounds are named as follows:

(K_0.5_Na_0.5_)NbO_3_ (KNN)
(K_0.5_Na_0.5_)_0.995_La_0.004_Nb_0.995_Mn_0.0045_O_3_ (KNNLM05)
(K_0.5_Na_0.5_)_0.992_La_0.008_Nb_0.990_Mn_0.0090_O_3_ (KNNLM1)
(K_0.5_Na_0.5_)_0.995_Eu_0.005_Nb_0.995_Fe_0.005_O_3_ (KNNEF05)
(K_0.5_Na_0.5_)_0.990_Eu_0.010_Nb_0.990_Fe_0.010_O_3_ (KNNEF1)

**Figure 1:**
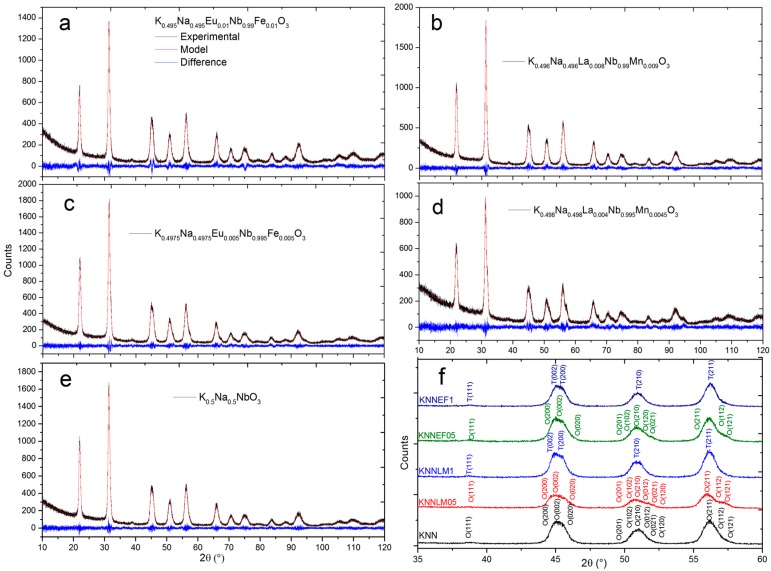
Experimental XRD patterns and results of their Rietveld refinement. (**a**) (K_0.5_Na_0.5_)_0.990_Eu_0.010_Nb_0.990_Fe_0.010_O_3_; (**b**) (K_0.5_Na_0.5_)_0.992_La_0.008_Nb_0.990_Mn_0.0090_O_3_; (**c**) (K_0.5_Na_0.5_)_0.995_Eu_0.005_Nb_0.995_Fe_0.005_O_3_; (**d**) (K_0.5_Na_0.5_)_0.995_La_0.004_Nb_0.995_Mn_0.0045_O_3_; (**e**) (K_0.5_Na_0.5_)NbO_3_; (**f**) comparison of some relevant peaks.

Figure 1 shows X-ray diffraction patterns and the calculated plots corresponding to the Rietveld refinement of the studied compounds. All of them show single crystalline phase indicating the solubility of the dopants into the KNN crystal structure (JCPDS No 00-061-0315), with evolution from the orthorhombic symmetry, described by Malic et al. [24], as the co-doping increase. The studied compositions were selected after a separate study, not shown here, to ensure that they constitute solid solutions. The solubility limit in KNNLM and KNNEF was determined to correspond to x = 0.054 for KNNLM and x = 0.056 for KNNEF samples. The obtained data from Rietveld analysis are listed in Table 1.

Table 1 shows the percentage of weighted profile R-factor (R_wp_), which is the figure of merit commonly used in Rietveld refinement, the crystal cell parameters, average crystal size and density of the samples. The comparison of the estimated crystal cell parameters and density for all samples with the data available in the literature for pure KNN revealed that these parameters are in accordance with those ones previously reported [24]. It is worth noting the changes in crystal parameters, which result in a change of crystal symmetry, from orthorhombic [25] to tetragonal (see the comparison of some relevant peaks enlarged in Figure 1) and finally a mixture of symmetries as the amount of co-doping increases.

**Figure 2:**
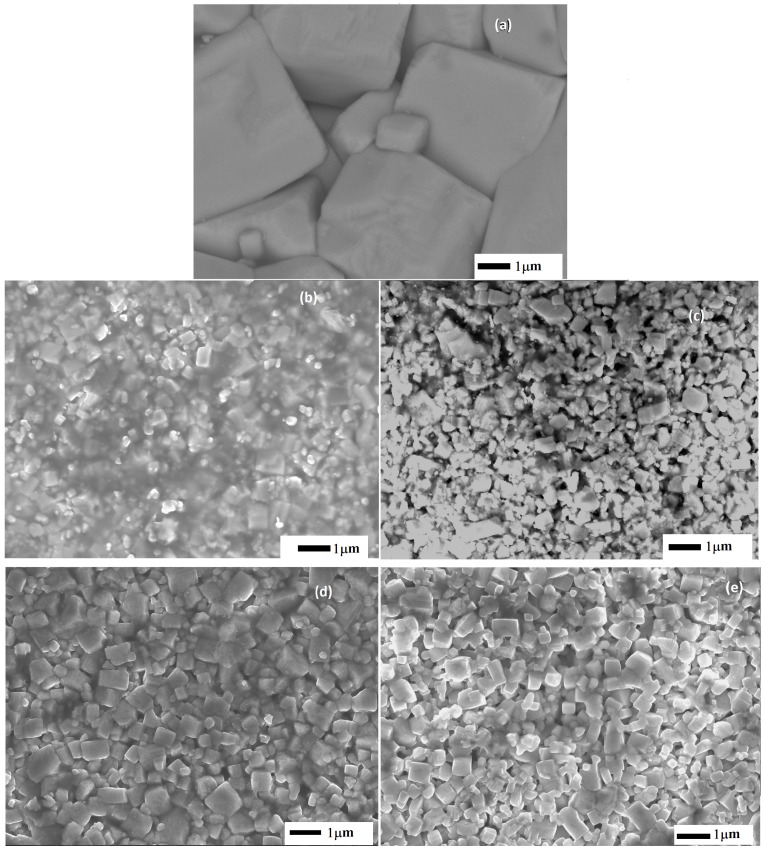
SEM images of sintered compounds (**a**) KNN T = 1105 °C; (**b**) KNNLM05 T = 1155 °C; (**c**) KNNLM1 T = 1155 °C; (**d**) KNNEF05 T = 1150 °C and (**e**) KNNEF1 T = 1135 °C.

SEM images of the higher density samples, sintered at different temperatures are shown in Figure 2. The main feature observed is the change in crystal size, which can be ascribed to the dopant i.e., its presence influences the inhibition of growth rates by changing the defect concentration and therefore the mass diffusivity at the grain surface [26].

Densification for sintered pellets is presented in Table 2, which were determined by comparing the calculated values from Rietveld refinement given in Table 1 and the measured by Arquimedes method.

In Table 2 the lower densification value was obtained for KNNLM1 sample, most probably due to structural defects associated with the difference in ionic size and oxidation state of the La^3+^ with respect to K^+^ and Na^+^ and Mn^2+^/Mn^4+^ with respect to Nb^5+^. In order to get insight on the defect structure and valence state of the dopants in the studied compounds both EPR and luminescence studies were carried out.

### 2.2. EPR and Optical Analysis

**Figure 3:**
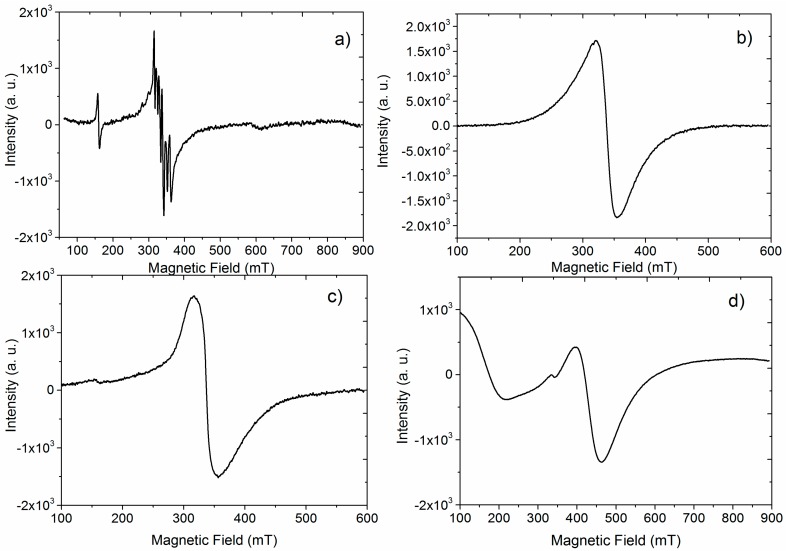
EPR spectra: (**a**) KNNLM05; (**b**) KNNLM1; (**c**) KNNEF05 and (**d**) KNNEF1.

EPR spectra are shown in Figure 3. The EPR technique is commonly used to determine the oxidation level of paramagnetic centers present in the compounds. The spectra of KNNLM05 (Figure 3a) and KNNLM1 (Figure 3b) exhibit classic Mn^2+^ transitions around 330 mT, which correspond to a ground state ^6^S_5/2_ with a spin value equal to S = 5/2. The difference between these two spectra is detailed as follows [27]. In Figure 3a, the spectrum is split in six lines due to the hyperfine interaction with the ^55^Mn nucleus [27,28,29,30,31,32]. In Figure 3b the spectrum shows only one broad signal, this response is related with the dipole–dipole interaction induced by the dopant concentration. This response is consistent with the presence of Mn^2+^ ion, which, at lower symmetries, could present the fine structure transition between the ligand field split in levels, corresponding to 5/2–3/2, 3/2–1/2, 1/2–−1/2, −1/2–−3/2 and −3/2–−5/2 transitions [28]. Besides, at lower symmetries of Mn^4+^ the fine structure is split in levels corresponding to 3/2–1/2, 1/2–−1/2 and −1/2–−3/2. However, as a result of large anisotropy only a central signal with its six hyperfine components due to Mn^2+^ was observed and this signal can be overlapped by the response of Mn^4+^, being more sensitive for Mn^2+^ [27,28,29,30,31,32,33,34,35].

Meanwhile, the spectrum for KNNEF05, given in Figure 3c, exhibits two transitions. The first one, with a small intensity at 154 mT might be assigned to Fe^3+^ by the 5/2–3/2 transition [36,37,38]. The second one at 330 mT, is more intense and corresponds to the Fe^3+^ ground state ^6^S_5/2_ due to the 1/2–−1/2 transition. Finally, the KNNEF1 compound (Figure 3d) shows two transitions: the first one at 330 mT and the second at 400 mT; these two signals could also be attributed to Fe^3+^. The particular shape of the spectrum may be due to a significantly distortion of the crystal environment around Fe^3+^ induced by the higher concentration of the dopant for this sample.

**Figure 4:**
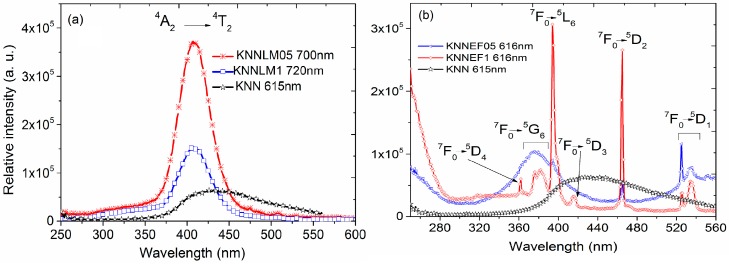
Excitation spectra of the (**a**) La–Mn co-doped KNN compounds and (**b**) Eu–Fe co-doped samples.

Luminescence excitation spectra are shown in Figure 4. The spectra of the solid solution co-doped with La-Mn (Figure 4a) exhibit a characteristic Mn^4+^ transition from ^4^A_2_–^4^T_2_ at 410 nm [39]. Whereas those of the compounds co-doped with Eu–Fe, (Figure 4b) exhibit several transitions: characteristic of f–f transitions of Eu^3+^, such as ^7^F_0_–^5^D_4_, ^7^F_0_–^5^G_6_, ^7^F_0_–^5^L_6_, ^7^F_0_–^5^D_3_, ^7^F_0_–^5^D_2_ and ^7^F_0_–^5^D_1_ [16,18] that are observed at 360 nm, 380 nm, 395 nm, 417 nm, 466 nm and 528 nm, respectively.

**Figure 5:**
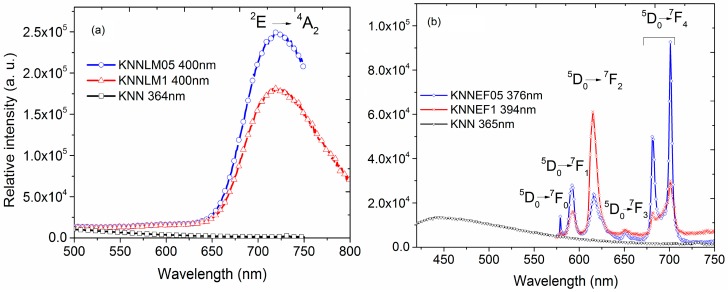
Emission spectra of (**a**) La–Mn co-doped KNN and (**b**) Eu–Fe solid solutions.

The emission spectra are presented in Figure 5. The spectra of the La-Mn co-doped compounds, (Figure 5a), exhibit a wide band centered at 720 nm, which may be ascribed to the ^2^E–^4^A_2_ transition of Mn^4+^ [39]. The emission spectra of solid solutions with Eu–Fe, (Figure 5b), show several transitions ascribed to Eu^3+^ f–f transition ^5^D_0_–^7^F_0_, ^5^D_0_–^7^F_1_, ^5^D_0_–^7^F_2_, ^5^D_0_–^7^F_3_ and ^5^D_0_–^7^F_4_ [16,18].

It should be noticed that Eu–Fe co-doped samples experiment a change in the concentration-dependent relative intensity of the ^5^D_0_–^7^F_1_ and ^5^D_0_–^7^F_2_ transitions. The relative intensity I(^7^F_2_)/I(^7^F_1_) of samples co-doped at 0.5% and 1% change from 0.86 to 3.77. This result is an evidence of a higher distortion in the crystal structure at the local level due to a higher dopant content, which explains the improvement of dielectric, ferroelectric and piezoelectric properties of the Eu–Fe co-doped compounds. Furthermore, this distortion correlates well with the Fe^3+^ environment showed from the EPR spectrum of sample KNNEF1.

**Figure 6:**
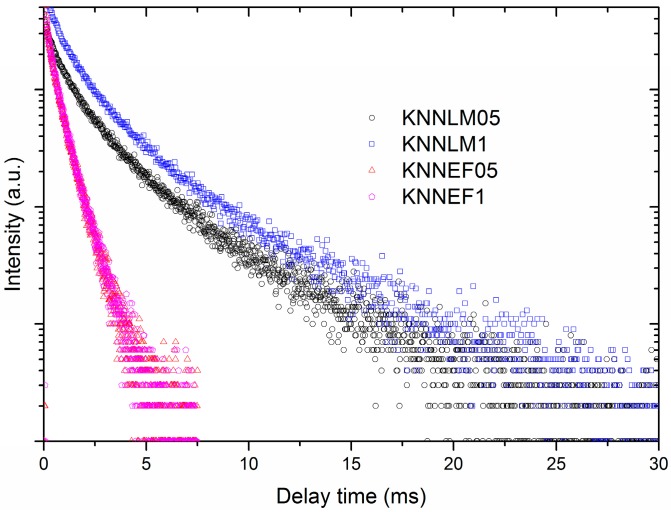
Luminescence decay curves of red emission for La–Mn and Eu–Fe co-doped KNN ceramics.

The luminescent decay curves for the co-doped samples are depicted in Figure 6. They all have features of a complex de-excitation dynamic, i.e., they show a non-exponential decay behavior [40]. This fact is expected because the optically active ions Mn^4+^/Eu^3+^ are settled into the perovskite structure at sites of K^+^ and Na^+^ (A-site) and Nb^5+^ (B-site), respectively, inducing the crystal structure distortion, and generating defects, which may be responsible for the implied dynamic de-excitation.

Additionally, we have calculated the average lifetime for the transitions of ^2^E–^4^A_2_ (Mn^4+^) and ^5^D_0_–^7^F_4_ (Eu^3+^) at different compositions. In the case of Eu^3+^, the average lifetime was 0.57 ms regardless of the concentration of Eu^3+^ and Fe^3+^. Similar results were obtained for the Mn^4+^: the average lifetimes were 2.25 ms and 2.2 ms for the samples doped at 0.5% and 1%, respectively. Large lifetime values may be related with ion transition, which is due to the parity and spin forbidden transition ^2^E–^4^A_2_ of Mn^4+^.

**Figure 7:**
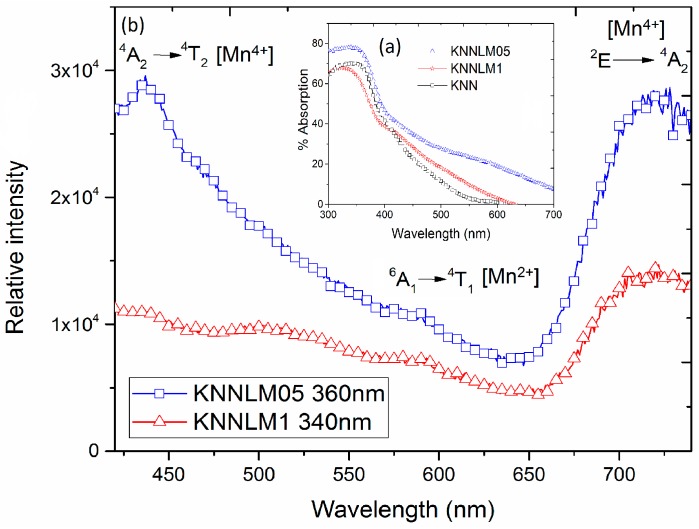
Absorption edge (**a**) and emission spectra; (**b**) for KNNLM05 and KNNLM1 compounds.

It should be noted that the excitation and emission spectra presented above do not show the presence of Mn^2+^ like in EPR study, for that case another kind of study must be carried out. The presence of Mn^2+^ can be determined by exciting the characteristic wavelength of the absorption edge (Figure 7a), which ensures the excitation of all the optical centers in the sample [36]. The absorption edge wavelengths for KNNLM05 and KNNLM1 compounds are 360 nm and 340 nm, respectively. These wavelengths were used to excite the compounds in order to obtain their emission spectra, showed in Figure 7b, which prove the existence of Mn^2+^ due to the presence of the band at 580 nm ascribed to the ^4^T_1_–^6^A_1_ Mn^2+^ transition [13]. Therefore, the presence of Mn^2+^ from the weak signal in Figure 7 matches with the results obtained in EPR. These results prove a mix of oxidation states inherent in Mn co-doped compounds, which may induce a conduction behavior at sufficiently high concentration by generation of lattice defects.

### 2.3. Electric Characterization

**Figure 8:**
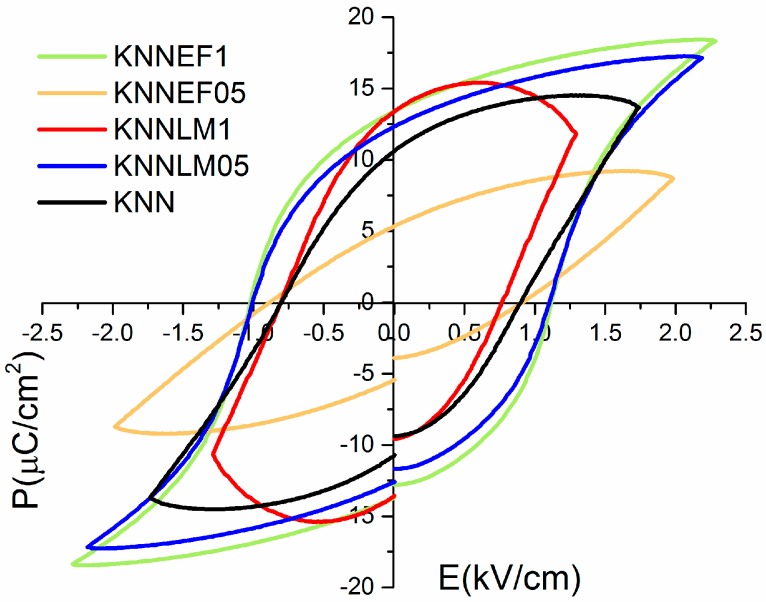
Ferroelectric loops of KNN and its solid solutions.

The ferroelectric loops acquired are presented in Figure 8. Most samples have saturated ferroelectric loops, except for the compounds KNNLM1 and KNNEF05. In the case of KNNLM1, the loop has a rounded shape, attributed to sample conductance. The conductance at room temperature result from manganese mixed valence state, borne out by EPR studies. However, KNNEF05 shows a subcoercive loop, thus, co-doping has caused an increase of the coercive field for this composition, most probably by introducing defect dipoles that stabilize the ferroelectric domains.

Table 3 shows the values of the remnant polarization (2Pr) and coercive field (2Ec) for all compounds. Regarding to coercive field, the solid solutions show lower values than un-doped KNN, whereas the value of remnant polarization increases and almost doubles the value observed for KNN. This behavior is similar to previous studies doped solely with transition metals [10,11,12], making these solid solutions soft ferroelectrics with a greater number of polarizable domains. 

**Figure 9:**
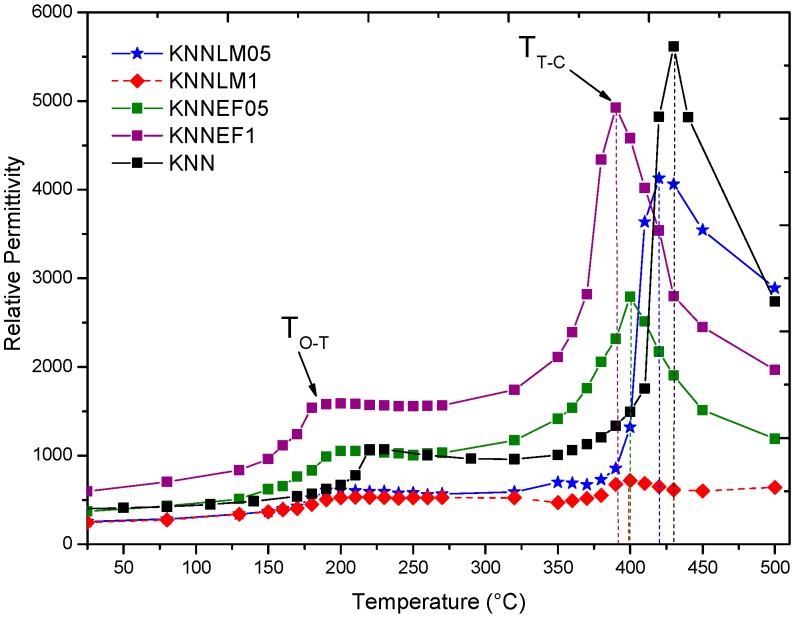
Dielectric permittivity vs. temperature, measured at 10 kHz, of KNN and its solids solutions.

The exhibited temperature dependence of the dielectric permittivity, measured at 10 kHz for sintered samples, given in Figure 9. The dielectric permittivity graphs show two phase transitions: T_O-T_ at 180–220 °C and T_T-C_ (T_Curie_) at 390–430 °C, depending on the compound used. For example, KNN presents the transitions at 220 °C and 430 °C, respectively, while KNNLM05 presents them at 195 °C and close to 420 °C. On the other hand, KNNEF1 shows the transitions at 180 °C and 390 °C, respectively.

**Figure 10:**
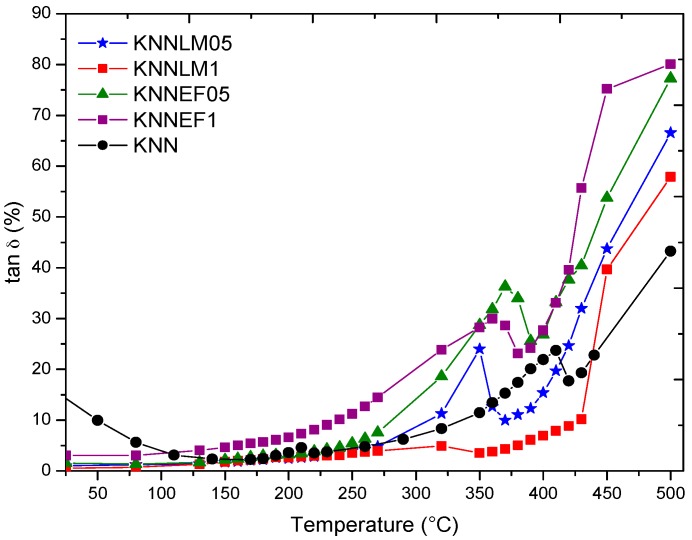
Dielectric loss tangent vs. temperature of KNN and its solid solutions, measured at 10 kHz.

Figure 10 shows the dependence of dielectric losses (tan *δ*) on the temperature measured at 10 kHz. Losses are low except for undoped KNN, most probably due to defects caused by the alkaline volatility that are avoided by co-doping [10,11,12].

The piezoelectric, elastic and dielectric parameters, obtained from the radial resonance of thickness poled thin disks analyzed using an iterative automatic method to obtain material coefficients with all losses [41], are shown in Table 4. The d_33_ value was also measured and d_h_ was calculated. It is noticeable that k_p_ values were improved for KNNLM05 composition, but if the dopant amount increases, the piezoelectric properties decrease. However, the KNNEF1 compound shows improved piezoelectric properties, but at higher dopant concentrations, values not shown here, the ferroelectric properties are lost.

**Figure 11:**
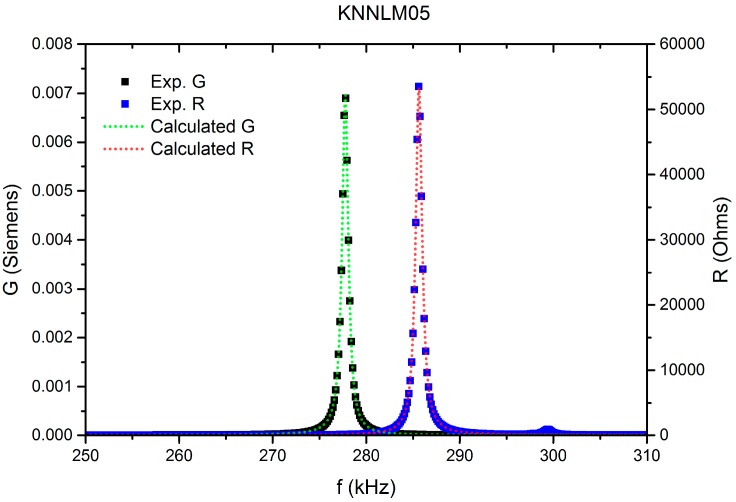
Planar resonance spectra for KNNLM05.

Figure 11 shows an example of the planar resonance spectra used for the calculation of the coefficients in Table 4. Instead of the usual plot of impedance modulus and phase the plot was made using the resistance (R = real part of the impedance) and conductance (G = real part of the admittance) peaks, since these are used in the calculation. It is noticeable the high agreement, also given by the regression factor (R^2^) (see Table 4), of the reproduced spectra (doted lines) to the measured one (symbols), which indicates the precision of the obtained materials parameters and the losses.

The best overall performances were obtained for KNNLM05 and KNNEF1 compounds. The slight decrease in some of the piezoelectric parameters (kp) for the compounds doped with manganese compared with the undoped KNN can be explained by the mixed valence states found by EPR studies. These mixed oxidation states produce a.c. conduction due to defects in the crystalline lattice of the composition with higher co-doping that result in duplicated dielectric tan *δ* in KNNLM1 with respect to undoped KNN. This effects are compensated with the better elastic properties of the co-doped ceramics, resulting from a fine grain dense microstructure, revealed by the higher modulus of the compliances (s_ij_^E,D^) and lower the stiffness (c_11p_^E,D^), while keeping similarly low mechanical losses, and ultimately in an improvement for some other parameters (d_33_ and d_h_).

However, in the compound co-doped with Eu–Fe the piezoelectric parameters decrease first in KNNEF05 and increase again in agreement with the doping effect of Fe^3+^ ions previously observed in the literature [14] and similar effects than in KNNLM concerning the elastic properties. 

The highest values in the co-doped ceramics for d_33_ (120 pC/N) and d_h_ (69 pC/N) were obtained for KNNLM05 and for k_p_ (31.1%) in KNNEF1 compounds, respectively. As it is well known, d_h_ is typically the most important parameter for underwater sound transducers, commonly manufactured using lead titanate ceramic material. Since KNNLM05 and KNNLM1 show the best d_h_ values, it proves to be suitable for sonar applications [42,43,44].

## 3. Materials and Methods

For the synthesis of KNN and the solid solutions (K_0.5_Na_0.5_)_1−(4x/5)_La_(4x/5)_Nb_1−x_Mn_4.5x/5_O_3_ and (K_0.5_Na_0.5_)_1−x_Eu_x_Nb_1−x_Fe_x_O_3_ (with x = 0.005 and 0.01), the following raw materials were used: Nb_2_O_5_ (99.9% Sigma-Aldrich, St. Louis, MO, USA), K_2_CO_3_ (99.8% Mallinckrodt, Phillipsburg, KY, USA), Na_2_CO_3_ (99.8% J.T. Baker, Xalostoc, Mexico), La(OH)_3_ (99.9% Sigma-Aldrich), Fe_2_O_3_ (99.77% Fisher Scientific, Fair Lawn, NJ, USA), Eu_2_O_3_ (99.9% Sigma-Aldrich) and MnO_2_ (98% Alfa Aesar, Ward Hill, MA, USA).

First, the carbonates and oxides were dried at 200 °C during 4 h before weighting. Then, stoichiometric amounts of the precursors were mixed in an agate mortar using acetone as dispersant. The mixture was calcined at 800 °C for 1 h, milled again in the agate mortar and heated at 950–1000 °C during 2 h. The powder was milled in a planetary ball mill using zirconia balls at 200 RPM for 10 h with ethanol as dispersant. Meanwhile, solid solutions with the highest dopant amount (solubility limit) were synthetized at 1000 °C for 8 h.

The powders were pressed into pellets of 13 mm diameter and 2 mm thickness at 45 MPa. The samples were conventionally sintered at temperatures between 1105 °C and 1150 °C for 2 h. Bulk densities of sintered ceramics was measured by Archimedes method. 

All samples were characterized by X-ray diffraction with Cu-Kα radiation (λ = 0.15418 nm, Bruker D8 Advance with a 0.0083 step size and 2 s integration time, Karlsruhe, Germany) and Rietveld refinements were carried out to determine the composition influence in the structure using TOPAS software (Bruker, Brisbane, Australia) [45]. The microstructures of sintered pellets were examined with a Scanning Electron Microscope (SEM, JEOL 7600F, Tokyo, Japan). Electron Paramagnetic Resonance (EPR) was carried out in an Electron Paramagnetic Resonance Spectrometer (JEOL JES-TE300, Tokyo, Japan), using a cylindrical cavity with the TE_011_ mode, operated at 100 kHz in *X* modulated band. The measurements were performed at room temperature in quartz tubes to determine the valence of paramagnetic centers in the obtained compounds. 

The excitation and emission spectra of the sintered pellets were carried out in a spectrometer (Edinburgh F900, Edinburg Instrument, Livingston, UK), whereas the absorption edge was acquired only for the compounds co-doped with La-Mn, using a spectrophotometer (Cary 5000, Agilent Technologies, San Jose, CA, USA).

For the luminescence decay curves of red emission. The average lifetime is defined as

(1) $$\tau_{m}=\int_{0}^{\infty }\mathrm{tI}\left(t\right)/\int_{0}^{\infty }I\left(t\right)$$

where I(t) is the intensity as a function of the time. This can be a also written as

$$\tau_{m}=\frac{\int_{0}^{\infty }\mathrm{tI}\left(t\right)}{\int_{0}^{\infty }I\left(t\right)}=\underset{i}{\sum }B_{i}\tau_{i}^{2}/\underset{i}{\sum }B_{i}\tau_{i}$$

We here determined the lifetime by fitting the experimental curves to the first two terms in the expression shown above.

In order to determine changes in dielectric and piezoelectric properties, sintered pellets were polished down to 1 mm of thickness, silver paste was applied and then annealed at 100 °C for 3 h. Dielectric and piezoelectric properties were measured using a Precision Impedance Analyzer (Agilent 4295A, San Jose, CA, USA).

The hysteresis loops (P-E) were acquired on a ferroelectric tester (Radiant RT66B work station, Radiant Technologies Inc., Alpharetta, CA, USA, at 100 Hz using an external 4 kV power supply source). For piezoelectric characterization, the samples were poled under 1–1.5 kV·mm^−1^ dc electric field at 175 °C for 30 min in a silicon oil bath. All measurements were performed after 24 h of poling process. The piezoelectric parameter d_33_ was measured with a d_33_ piezometer system (PM300-PIEZOTEST). The d_31_ parameter, as well as the electrochemical coupling factors k_P_ and k_31_, and g_31_ parameter, together with elastic constants and permittivity at the resonance frequency, were calculated using the resonance method by an automatic iterative analysis method of the complex impedance at the radial mode of thin disks, thickness poled [41].

## 4. Conclusions

(K_0.5_Na_0.5_)_1−(4x/5)_La_(4x/5)_Nb_1−x_Mn_4.5x/5_O_3_ and (K_0.5_Na_0.5_)_1−x_Eu_x_Nb_1−x_Fe_x_O_3_ solid solutions (with x = 0.005 and 0.01) were successfully synthesized by solid-state method and sintered to obtain dense ceramics. Pure perovskite phase was achieved for all compositions as observed by X-ray diffraction. The Rietveld refinements show shifts in lattice parameters and result in a change of crystal symmetry, from orthorhombic to tetragonal, as the amount of co-doping increases.

EPR and optical measurements confirm the oxidation state of the Mn ions: the wide band centered at 720 nm, ascribed to the ^2^E–^4^A_2_ transition of Mn^4+^ of the emission spectra, and the presence of the band at 580 nm ascribed to the ^4^T_1_–^6^A_1_ transition, due to the Mn^2+^ from excitation at the absorption edge. The mixed oxidation states of manganese explain the conductive behavior at high dopant concentrations, resulting in leaky hysteresis loops and reduction of the piezoelectric coefficients. 

EPR results and the ^4^T_1_–^6^A_1_ transition at 705 nm ascribed to Fe^3+^ of the emission spectra; confirm the oxidation state of the Fe^3+^ ions. The change in the relative intensity of the transitions I (^7^F_2_)/I(^7^F_1_) of samples of the solid solution with Eu–Fe is an evidence of a higher distortion in the crystal structure due to the dopant content, which is directly related with the dielectric, ferroelectric and piezoelectric properties of the Eu–Fe solid solution.

Generation of optical properties, while piezoelectric properties are kept similar to pure KNN), and higher for hydrostatic applications in KNNLM, were exhibited by the La–Mn co-doped samples especially at 0.5% mol (KNNLM05). Meanwhile for Eu–Fe co-doped samples, the best electromechanical properties were obtained at 1 mol % (KNNEF1).

## Figures and Tables

**Table 1:** Crystallographic parameters obtained from Rietveld refinement analysis for all solid solutions.

Composition	KNN	KNNLM05	KNNLM1	KNNEF05	KNNEF1
R_WP_ %	10.03	11.07	10.09	9.76	11.04
a (Å)	4.005 ^a^	3.101 (6)	3.964 (2)	3.261 (4)	3.976 (2)
b (Å)	3.944 ^a^	3.601 (2)	3.964 (3)	3.317 (4)	3.976 (2)
c (Å)	4.002 ^a^	4.005 (6)	3.989 (2)	4.005 (4)	3.981 (3)
Crystal system	ortho	ortho	tetragonal	ortho	tetragonal
Space group	Amm2	Amm2	P4mm	Amm2	P4mm
Volume (Å^3^)	127 (2)	121 (1)	122 (1)	122 (1)	124 (1)
Average crystallite size (nm)	37 (3)	54 (4)	8 (3)	39 (3)	30 (2)
Calculated density (g/cm^3^)	4.578	4.495 (6)	4.528 (3)	4.543 (4)	4.568 (4)

^a^ Value taken from the reference [24].

**Table 2:** Theoretical and experimental density and densification percentage for sintered compounds.

Compound	Calculated Density (g/cm^3^)	Experimental Density (g/cm^3^)	Densification (%)
KNN	4.578	4.329	94.6
KNNLM05	4.495 (6)	4.399	97.9
KNNLM1	4.528 (3)	4.266	94.2
KNNEF05	4.543 (4)	4.378	96.4
KNNEF1	4.568 (4)	4.419	96.7

**Table 3:** Remnant polarization (2P_r_) and the coercive field (2E_c_) for sintered ceramics.

Composition	2P_r_ (μC/cm^2^)	2E_C_ (kV/cm)
KNN	6.2	0.9
KNNLM05	12.45	1.05
KNNEF1	13.54	1.07

**Table 4:** Piezoelectric, elastic and dielectric coefficients including all losses and other relevant parameters at the radial resonance of a thin disk, thickness poled, and measured at the d_33_-meter of KNN and its solid solutions.

Sample	KNN	KNNLM05	KNNLM1	KNNEF05	KNNEF1
*δ* (g/cm^3^)	4.34	4.4	4.27	4.375	4.415
R^2^	0.9973	0.9978	0.9992	0.9982	0.9809
Np (kHz·mm)	3357	2997	2353	2874	3378
kp (%)	34.1	26,7	16.8	23.9	31.9
k_31_ (%)	20.0	13.7	12.0	14.2	18.7
Poisson’s ratio	0.311 + 0.0003 i	0.475 + 0.0001 i	–	0.295 − 0.0001 i	0.312 + 0.0001 i
c_11p_^E^ (10^10^ N·m^−2^)	11.43 + 0.04 i	8.42 + 0.02 i	7.03 + 0.04 i	8.52 + 0.04 i	11.76 + 0.16 i
s_11_^E^ (10^−12^ m^2^·N^−1^)	9.69 − 0.03 i	15.33 − 0.04 i	14.23 − 0.07 i	12.85 − 0.05 i	9.42 − 0.13 i
s_12_^E^ (10^−12^ m^2^·N^−1^)	−3.02 + 0.01 i	−7.28 + 0.02 i	–	−3.79 + 0.02 i	−2.94 + 0.04 i
d_31_ (10^−12^ C·N^−1^)	−28.99 + 0.27 i	−25.55 + 0.19 i	−19.20 + 0.36 i	−30.09 + 0.44 i	−40.13 + 1.56 i
ε_33_^T^ (real)	244.86	257.38	203.14	395.23	552.15
tan *δ*	0.015	0.012	0.030	0.021	0.037
s_66_^E^ (10^−12^ m^2^·N^−1^)	25.41 − 0.09 i	45.23 − 0.13 i	27.72 − 0.14 i	33.26 − 0.14 i	24.71 − 0.33 i
c_11p_^D^ (10^10^ N·m^−2^)	12.41 + 0.04 i	8.90 + 0.03 i	7.13 + 0.04 i	8.86 + 0.04 i	12.64 + 0.15 i
s_11_^D^ (10^−12^ m^2^·N^−1^)	9.30 − 0.03 i	15.05 − 0.04 i	14.02 − 0.07 i	12.59 − 0.0520 i	9.09 − 0.11 i
s_12_^D^ (10^−12^ m^2^·N^−1^)	−3.40 + 0.01 i	−7.60 + 0.02 i	–	-4.04 + 0.02 i	−3.27 + 0.05 i
g_31_ (10^−3^ m·V·N^−1^)	−13.37 − 0.08 i	−11.21 − 0.06 i	−10.67 − 0.14 i	−8.60 − 0.05 i	−8.21 + 0.01 i
d_33_ (10^−12^ C/N)	98	120	94	105	116
d_h_ (10^−12^ C/N)	40	69	56	45	36
